# Temperature-Inducible Transgenic *EDS1* and *PAD4* in *Arabidopsis* Confer an Enhanced Disease Resistance at Elevated Temperature

**DOI:** 10.3390/plants10061258

**Published:** 2021-06-21

**Authors:** Junchen Leng, Weishan Tu, Yanbing Hou, Haitao Cui

**Affiliations:** Key Laboratory of Ministry of Education for Genetics, Breeding and Multiple Utilization of Crops, Plant Immunity Center, Fujian Agriculture and Forestry University, Fuzhou 350002, China; 3180130011@fafu.edu.cn (J.L.); weishan@mail.ustc.edu.cn (W.T.); houyanbing@mail.ustc.edu.cn (Y.H.)

**Keywords:** temperature, disease resistance, immunity, EDS1, PAD4

## Abstract

Temperature is one of the most important environmental factors greatly affecting plant disease development. High temperature favors outbreaks of many plant diseases, which threaten food security and turn to be a big issue along with climate change and global warming. Here, we found that concurrent constitutive expression of the key immune regulators *EDS1* and *PAD4* in *Arabidopsis* significantly enhanced resistance to virulent bacterial pathogen *Pseudomonas syringae* pv. *tomato* at elevated temperature; however, autoimmunity-related growth retardation was also observed on these plants at a normal temperature. To balance this growth-defense trade-off, we generated transgenic plants dual expressing *EDS1* and *PAD4* genes under the control of a thermo-sensitive promoter from the *HSP70* gene, whose expression is highly induced at an elevated temperature. Unlike constitutive overexpression lines, the proHSP70-EP transgenic lines exhibited enhanced resistance to bacterial pathogens at an elevated temperature without growth defects at normal condition. Thus, this study provides a potential strategy for genetic manipulation of plants to deal with the simultaneous abiotic and biotic stresses.

## 1. Introduction

Plant diseases are one of the most important causes leading to crop losses; thus, the broad diversity and rapid evolution of pathogens bring a big challenge for modem plant breeding. To defend against pathogens, plants have evolved a sophisticated innate immune system, which recognizes infectious microbes and activates defense responses mainly through two types of receptors: (1) one is the cell surface localized pattern recognition receptors (PRRs) that perceive pathogen-associated molecular patterns (PAMPs) and activate immune responses, which is called PAMP-triggered immunity (PTI); (2) the other type is intracellular nucleotide-binding/leucine-rich-repeat (NLR) receptors that intercept pathogen effectors and induce a robust disease response called effector-triggered immunity (ETI) [1,2]. Both PTI and ETI induce similar immune responses, including transient calcium influx, a rapid production of reactive oxygen species, activation of mitogen-activated protein kinase (MAPK) cascades and defense-related transcriptional reprograming [1]. The immune responses have to be tightly controlled, as prolonged or auto-activated immune responses result in plant growth retardation and cell death [3]. Due to intensive studies on the plant immune system, an increasing number of immune regulators have been identified, and their molecular mechanisms are being uncovered. Several immune regulators have been applied in breeding for resistant crops with expected benefits [4,5].

Ambient environment is one of the key determinants for plant disease epidemics [6]. Humidity and temperature are major climate factors that have profound impact on the outcomes of plant–microbe interactions, as the continuous hot and humid weather is prone to outbreaks of many plant diseases [7]. How these specific environmental factors affect the host and the pathogen as well as their interactions is an important question to be addressed and will be very helpful for making new strategies in plant breeding. It has been shown that high temperatures significantly increase *Arabidopsis* susceptibility to bacterial pathogen *Pseudomonas syringae* pv. *tomato* DC3000 (*Pst* DC3000) [8,9]. *Pst* causes the bacterial speck disease on tomato leaves which has been used as a model for studying plant–bacterial interactions [10]. The high temperature-provoked disease susceptibility is associated with boosted PTI but reduced ETI in plants, consisting of the reports that several NLRs-mediated resistances are abolished at elevated temperatures [11,12,13,14]. Recently, Huot et al. discovered that an elevated temperature enhances disease susceptibility at least partially by suppressing biosynthesis of salicylic acid (SA), an essential phytohormone involved in plant resistance to biotrophic and semi-biotrophic pathogens [8]. Moreover, application of benzothiadiazole (BTH), an SA synthetic analogue, could provide enhanced disease resistance at elevated temperatures [8]. This provides a useful clue regarding the genetic manipulation of the SA-related plant immune system to enhance disease resistance at elevated temperatures.

Plant NLR receptors can be classified into two major groups depending on their distinct N-terminal domains: coiled-coil-NB-LRRs (CNLs) and Toll/interleukin-1 receptor (TIR)-NB-LRRs (TNLs). Enhanced disease susceptibility 1 (EDS1) is a key signaling component in TNL-mediated immunity and also serves as an important positive regulator in SA synthesis [15]. EDS1 is required for all tested TNLs-mediated ETI and for basal resistance to virulent pathogens [3,15]. *Pst* DC3000-induced SA accumulation is dramatically compromised in *Arabidopsis eds1* mutant plants [16]. EDS1 interacts with its sequence-related protein phytoalexin deficient 4 (PAD4) and senescence-associated gene 101 (SAG101) forming a heteromeric regulatory unit for signaling [16,17,18]. Thus, overexpression of EDS1 or PAD4 alone does not render *Arabidopsis*-enhanced resistance to *Pst* DC3000, probably due to less amounts of their interacting partners [19]. Indeed, co-overexpression of EDS1 and PAD4 lead to autoimmunity and enhanced disease resistance [19]. Transcriptional profile analysis revealed that EDS1-PAD4 not only promotes expression of the *isochorismate synthase 1* (*ICS1*) gene, encoding the key enzyme for pathogen-induced SA biosynthesis, but also a great portion of the SA-responsive genes in parallel with SA accumulation [19]. In addition, EDS1-PAD4 could boost SA pathways through antagonizing jasmonic acid (JA) signaling pathway by suppressing the master transcription factor MYC2 [20].

Since EDS1-PAD4-mediated immunity could bypass SA accumulation to induce expression of SA-responsive genes [19], we wondered whether the stimulation of EDS1-PAD4 signaling could compensate for the loss of SA synthesis at elevated temperatures. In this study, we examined the EDS1-PAD4-mediated immune functions and tested their potential application in enhancing plant resistance at elevated temperatures.

## 2. Results

### 2.1. The Autoimmunity Activated by Dual Overexpression of EDS1 and PAD4 Is Not Suppressed at Elevated Temperatures

**Figure 1:**
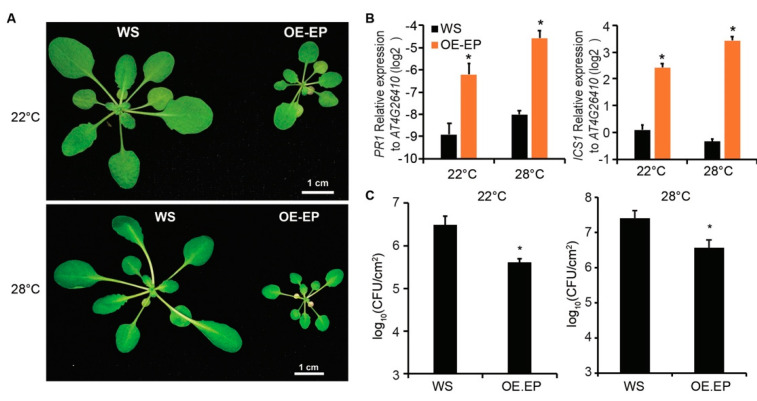
Dual overexpression of *EDS1* and *PAD4* in *Arabidopsis* enhances resistance to bacterial pathogens at normal and elevated temperatures. (**A**) Developmental phenotype of wild-type *Arabidopsis* ecotype WS and transgenic line constitutively expressing *EDS1* and *PAD4* driven by 35S promoter (OE.EP) grown at 22 or 28 °C. When photographed, plants were 4 weeks old, grown at 22 °C or 3 weeks old grown at 28 °C. Bars = 1 cm. (**B**) Expression of salicylic acid pathway genes *PR1* and *ICS1* in leaves of plants in (a) measured by quantitative reverse transcription-polymerase chain reaction (qRT-PCR). Gene expression was normalized to *AT4G26410*. Error bars represent mean ± SD of three biological replicates. * indicates significant difference to WS in a Student’s *t*-test (*p* < 0.05). (**C**) Growth of bacterial strain *Pst* DC3000 in leaves of WS or OE.EP plants. Leaves of 4-week-old (22 °C or 3-week-old (28 °C) plants were hand-infiltrated with bacterial suspensions (OD600 = 0.0002) and bacterial titers were determined at 3 dpi. Error bars represent mean ± SD of six biological replicates. * indicates significant difference to WS in a Student’s *t*-test (*p* < 0.05). The above experiments were repeated three times with similar results.

Previously, we have shown that dual overexpression of *EDS1* and *PAD4* in *Arabidopsis* (OE.EP) results in autoimmunity and enhances resistance to a virulent bacterial pathogen *Pst* DC3000 at a normal temperature (22 °C) [19]. In *Arabidopsis,* many TNL-mediated immunities are suppressed at an elevated temperature (28 °C) [9]. Because EDS1 and PAD4 function downstream of TNLs [3,15], we wondered whether the autoimmunity in the OE.EP line is temperature dependent. To test this, we grew OE.EP plants at 28 °C. We found that the OE.EP plants exhibited a dwarf phenotype at both 22 and 28 °C compared with wild-type (WT) ecotype Wassilewskija-2 (Ws) (Figure 1A). Consistently, the expressions of the SA synthesis gene *ICS1* and the defense marker gene *PR1* in OE.EP plants were much higher than in WT plants in both conditions (Figure 1B), indicating that the enhanced SA pathway in OE.EP plants was maintained at an elevated temperature. Moreover, the growth of *Pst* DC3000 in OE.EP plants was more than ten times less compared with that in WT plants at 28 °C (Figure 1C), indicating that OE.EP plants are more resistant to *Pst* DC3000 than WT plants at an elevated temperature. These data demonstrate that the autoimmunity mediated by dual overexpression of *EDS1* and *PAD4* is not suppressed at an elevated temperature.

### 2.2. Selection of Promoters That Are Induced at Elevated Temperature

Constitutive dual overexpression of *EDS1* and *PAD4* enhances plant resistance, but also results in autoimmunity-related growth retardation, which limits its application in plant breeding for disease resistance. However, the EDS1-PAD4-mediated resistance is insensitive to an elevated temperature (Figure 1), which provides potential gene resources for enhancing plant resistance at an elevated temperature in plant breeding. Thus, a method is needed to control immune activity of EDS1-PAD4 regulon at a normal temperature but reinforce their activity at an elevated temperature. For this, we sought to use a temperature-responsive promoter to drive the expression of *EDS1* and *PAD4*. Such promoter activity should be relatively low at a normal temperature but drive high expression of *EDS1* and *PAD4* at an elevated temperature.

**Figure 2:**
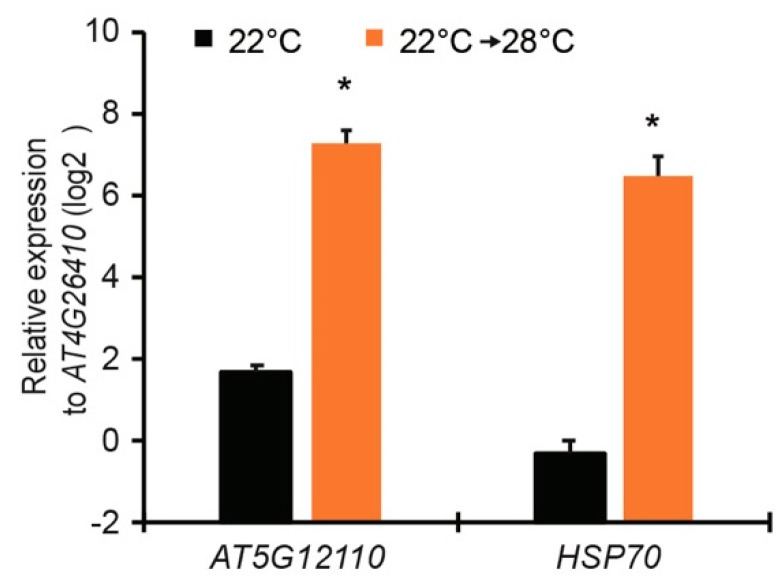
Quantitative expression of *AT5G12110* and *HSP70* genes in leaves of *Arabidopsis* wild-type Col plants shifted from 22 to 28 °C for 24 h. Gene expression was measured by quantitative reverse transcription polymerase chain reaction (qRT-PCR) and normalized to housekeeping gene *AT4G26410*. Error bars represent mean ± SD of three biological replicates. * indicates significant difference to 22 °C in a Student’s *t*-test (*p* < 0.05).

To select promoters which fulfill the above criteria, we analyzed published microarray dataset (GSE50019) and RNA-seq dataset (GSE80448) of *Arabidopsis* plants shifted from 28 to 19 °C [21,22]. The genes with high expression level at 28 °C and low expression level when shifted to 19 °C were selected (Table 1 and Table 2). Among those genes, *AT5G12110* and *AT3G12580* (*HSP70*) were listed on top with a high expression level at 28 °C in both datasets. We then verified the expression of these two genes in wild-type Col-0 plants shifted from 22 to 28 °C. Consistent with the microarray and RNA-seq data, the transcripts of *AT5G12110* and *HSP70* genes were highly induced when plants were shifted to 28 °C for 24 h (Figure 2). Thus, promoters of *AT5G12110* and *HSP70* were cloned to drive expression of both *EDS1* and *PAD4* genes in new transgenic lines.

### 2.3. Construction of Transgenic Lines Harboring Temperature-Inducible EDS1 and PAD4

**Figure 3:**
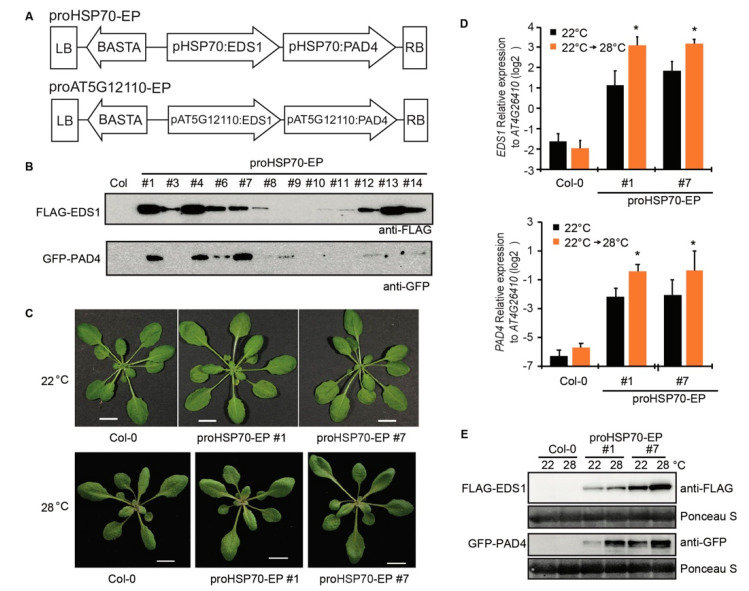
Construction of transgenic *Arabidopsis* Col lines expressing inducible *EDS1* and *PAD4* at elevated temperatures. (**A**) The diagrams show arrangement of *BASTA, EDS1,* and *PAD4* genes within left border (LB) and right border (RB) on the two T-DNA binary constructs. pHSP70: the promoter of *HSP70*, pAT5G12110: the promoter of *AT5G12110*. (**B**) Immunoblots probed with anti-FLAG and anti-GFP antibodies show FLAG-EDS1 and YFP-PAD4 proteins accumulation in T1 transgenic *Arabidopsis* lines expressing *FLAG-EDS1* and *YFP-PAD4* under the control of promoter of *HSP70* (proHSP70-EP) with WT Col as control. (**C**) Developmental phenotype of 4-week-old (22 °C) and 3-week-old (28 °C) plants of T3 homozygous lines of proHSP70-EP (line #1 and #7). Bars = 1 cm. (**D**) Expression of *EDS1* and *PAD4* in leaves of Col and proHSP70-EP lines shifted from 22 to 28 °C for 24 h measured by qRT-PCR. Gene expression was normalized to *AT4G26410*. Error bars represent mean ± SD of three biological replicates. * indicates significant difference to 22 °C in a Student’s *t*-test (*p* < 0.05). (**E**) Immunoblots probed with anti-FLAG and anti-GFP antibodies show FLAG-EDS1 and YFP-PAD4 protein accumulation in T3 homozygous lines of proHSP70-EP (line #1 and #7) shifted from 22 to 28 °C for 24 h. Ponceau staining of the blots indicates the equal loading.

Using Goldengate cloning system, we made two plasmids containing the *BASTA* gene for Basta resistance, N-terminal tagged *FLAG-EDS1,* and *GFP*-*PAD4.* Both *FLAG-EDS1* and *GFP-PAD4* were driven by the 2 kb promoter of *AT5G12110* on one plasmid, and by the 2 kb promoter of *HSP70* on the other plasmid (Figure 3A). The two plasmids were transformed into *Agrobacteria* GV3101 and then the transgenic *Arabidopsis* lines were generated using *Agrobacteria*-mediated transformation. The transgenic lines were named as proHSP70-EP (promoter of *HSP70* driving *EDS1* and *PAD4*) and proAT5G12110-EP (promoter of *AT5G12110* driving *EDS1* and *PAD4*), respectively. The protein accumulation of FLAG-EDS1 and GFP-PAD4 in individual T1 lines was examined by immunoblotting. The FLAG-EDS1 and GFP-PAD4 proteins could be detected in several proHSP70-EP lines (Figure 3B), but FLAG-EDS1 was undetectable in all proAT5G12110-EP lines for unknown reason (Figure S1). Thus, we focused on the proHSP70-EP lines in the further studies. Two proHSP70-EP T3 homozygous lines were obtained, which did not show any growth defect either at a normal temperature or at an elevated temperature (Figure 3C). The expression level of *EDS1* and *PAD4* genes in proHSP70-EP lines was increased when plants were transferred from 22 to 28 °C for 24 h (Figure 3D). Consistent with the increased transcripts, the FLAG-EDS1 and GFP-PAD4 proteins accumulated in proHSP70-EP lines at 28 °C compared with those at 22 °C (Figure 3E). These data show that we have successfully obtained the transgenic lines expressing inducible *EDS1* and *PAD4* at an elevated temperature.

### 2.4. Temperature-Controlled Expression of EDS1 and PAD4 Confers Enhanced Resistance to Bacterial Pathogens at Elevated Temperatures

**Figure 4:**
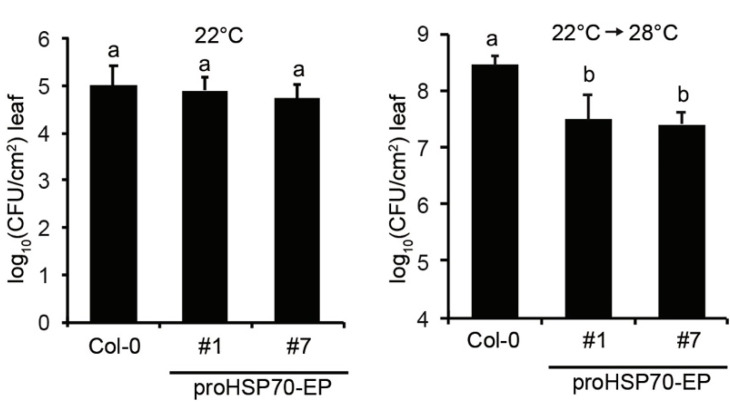
Temperature-inducible *EDS1* and *PAD4* confer enhanced resistance to bacterial pathogens at elevated temperatures. Growth of bacterial strain *Pst* DC3000 in leaves of *Arabidopsis* Col and transgenic lines expressing *FLAG-EDS1* and *YFP-PAD4* under control of the promoter of *HSP70* gene (proHSP70-EP). Plants were grown at 22 °C for 4 weeks, then half of them were shift to 28 °C 24 h before bacterial infection for measuring resistance at an elevated temperature. Plant leaves were hand-infiltrated with bacterial suspensions (OD600 = 0.0002) and bacterial titers were determined at 3 dpi. Error bars represent mean ± SD of six biological replicates. Different letters indicate statistical significance (*p* < 0.05) determined by one-way ANOVA followed by Tukey’s HSD. The experiment was repeated three times with similar results.

To examine whether temperature-controlled expression of *EDS1* and *PAD4* in proHSP70-EP lines confers enhanced pathogen resistance at elevated temperature, we tested *Pst* DC3000 resistance on proHSP70-EP plants shifted from 22 to 28 °C 24 h before pathogen infection. The results showed that proHSP70-EP lines exhibited WT-like resistance to *Pst* DC3000 at 22 °C, while they showed significantly enhanced resistance to *Pst* DC3000 at 28 °C (Figure 4). This result agrees that transgenes of *EDS1* and *PAD4* under the control of the temperature-responsive promoter could enhance resistance to bacterial pathogens at elevated temperatures in *Arabidopsis.*

## 3. Discussion

Elevated temperature has long been shown to promote disease in many plant-pathosystems. To date, genes which can enhance plant disease resistance at elevated temperatures have been rarely reported. Here, we show that dual overexpression of *EDS1* and *PAD4* in *Arabidopsis* enhances plant resistance to virulent bacterial pathogen *Pst* DC3000. High temperatures do not affect the high expression of SA synthesis gene *ICS1* in OE.EP plants (Figure 1), indicating that activated EDS1-PAD4 regulon restores SA biosynthesis which is suppressed at elevated temperatures. Previous studies have shown that the EDS1-PAD4 complex functions closely related with TNLs. For example, EDS1 has been shown to associate with paired TNLs RPS4/RRS1, that recognizes bacterial effector AvrRps4 and PopP2 for ETI activation [23,24]. Overexpression of RPS4 also results in EDS1-dependent autoimmunity, which is totally abolished at elevated temperature. In this study, we found that an elevated temperature did not break down the autoimmunity mediated by dual overexpression *EDS1* and *PAD4* (Figure 1), supporting that EDS1-PAD4 functions downstream of RPS4. This also suggests that an elevated temperature suppresses RPS4-mediated autoimmunity by affecting EDS1-PAD4 protein accumulation or other components upstream of EDS1, likely the TNL receptor itself. Consistent with this suspicion, it has been shown that an elevated temperature suppresses TNL receptor SNC1-mediated ETI through affecting its nuclear localization, which is essential for SNC1 activating immune responses [12].

It has been reported that *EDS1* and *PAD4* are prerequisites for several autoimmunity mutants, such as *snc1, bon1*, and *cpr1* [11,12,25]. The constitutive defense phenotypes of the *Arabidopsis constitutive induced resistance 1* (*cir1*) mutant require both *EDS1* and *PAD4* [26]. The enhanced resistance to *Pst* DC3000 in *cir1* was abolished at 25 °C [26]. The *CIR1* gene has not been cloned yet. It is possible that *cir1* is a gain function mutation of an TNL gene, such as *snc1*, whose autoimmunity requires EDS1 [27], and is suppressed by an elevated temperature (28 °C) [13], or that the *cir1* mutant triggers activation of an TNL receptor.

Plant defense activation generally comes at the expense of plant growth [28]. The growth–defense trade-off is the one of the major issues that should be considered in plant breeding for biotic and abiotic resistance. Overexpression of positive regulators in plants enhances disease resistance, but is usually associated with a significant fitness cost [29,30], which limits the application of this approach in the practice of crop breeding. For example, overexpression of the SA receptor *NRP1* in rice enhances plant resistance to blast disease, but also results in dwarf plants and poor yield [31]. Dual overexpression of *EDS1* and *PAD4* also results in autoimmunity-related growth retardation [19]. To overcome the growth–defense trade-off mediated by overexpression of *EDS1* and *PAD4*, we sought a more stringent way by expressing *EDS1* and *PAD4* genes under the control of temperature in transgenic plants. From published transcriptome datasets, we identified promoters of *AT5G12110* and *HSP70* that were highly induced at an elevated temperature. We successfully attained transgenic plants expressing *EDS1* and *PAD4* genes driven by promoter of *HSP70* (proHSP70-EP)*,* and both proteins were induced at an elevated temperature as expected. The proHSP70-EP plants growing at a normal temperature developed normally as seen in WT plants (Figure 3C). In pathogen infection assays, the proHSP70-EP plants exhibited enhanced resistance to *Pst* DC3000 (Figure 4), demonstrating this approach works in the model plant species. EDS1 has been documented to be a conserved key components in TNL ETI in dicots [15], including many important Solanaceous food plants [32]. Further study could be worth for application on the food plants, such as pepper, potato and tomato, to enhance disease resistance at an elevated temperature. Thus, our study provides a potential strategy for genetic engineering of environment-relevant components in plants to enhance disease resistance facing global warming.

## 4. Materials and Methods

### 4.1. Plant Materials, Growth Conditions and Pathogen Strains

*Arabidopsis thaliana* wild-type accessions used in this research are Wassilewskija-2 (Ws) and Columbia-0 (Col). Transgenic OE.EP line was published in Cui et al., 2017 [19]. *Pseudomonas syringe* pv. *tomato* (*Pst*) strain DC3000 was maintained as previously described [19]. The seeds were nursed in MS (Murashige and Skoog) medium and then were transplanted into soil after 1 week. All plants were grown in a growth chamber setting as 22 (normal) or 28 °C (elevated) and 65% relative humidity, with a 9 h/15 h light/dark period and the light intensity was 150 iE/m^2^s.

### 4.2. Golden Gate Cloning and Generation of Arabidopsis Transgenic Lines

Promoters (2 kb upstream of start codon) of *AT5G12110* and *HSP70* were amplified by PCR and cloned into the level 0 vector pAGM1251, respectively. *EDS1* and *PAD4* coding sequence were cloned into the level 0 vector pICH41308. At level 1 vectors, *EDS1* were cloned into pICH47811 (*proAT5G12110: or proHSP70:3xFLAG-EDS1-35S_term*), and *PAD4* were cloned into pICH47822 (*proAT5G12110: or proHSP70:YFP-PAD4-35S_term*). For level 2 constructs in pAGM4673, the *pNos:BASTA^R^-Nos_term*—cassette was placed at position 1, *EDS1*—at position 2, and *PAD4*—at position 3. Backbones, tags, and the terminator module, as well as the BASTA^R^ expression cassette, are from the Golden Gate cloning toolkit [13]. Primers used for cloning are provided in Supplemental Table S1.

The level 2 constructs were transformed into agrobacterium strain GV3101 and used to transform *Arabidopsis* plants. The transformants growing in soil were selected after spraying with 0.01% BASTA (Sangon, #A614229).

### 4.3. Pathogen Infection Assays

For bacterial growth assays, *Pst* DC3000 (OD_600_ = 0.0002) in sterilized water were hand-infiltrated into leaves of 4-week-old plants, and bacterial titers were measured as previously described [17]. Statistical analysis of bacterial growth data was done by student’s *t*-test.

### 4.4. RNA Analysis

Total RNA from leaves of 4-week-old plants was extracted using TRIzol Regent (Invitrogen, #15596018), and then cDNA was obtained using RNA as a template by GoScriptTM Reverse Transcription kit (Promega, #M170B). qRT-PCR analysis was performed on a CFX Connect machine (Biorad). Expression of the test genes were normalized to *AT4G26410*, as described previously [19].

### 4.5. Protein Extraction and Immunoblotting

Leaves from 4-week-old plants were frozen in liquid nitrogen and ground by TissueLyser (Qiagen). Then the total proteins were extracted in extraction buffer (50 mM of Tris pH7.5, 150 mM of NaCl, 10% (*v*/*v*) glycerol, 2 mM of EDTA, 5 mM of DTT, 0.1% Triton X-100, protease inhibitor cocktail (Roche, 1 tablet per 50 mL)). Lysates were centrifuged for 15 min at 14,000 rpm at 4 °C. After adding the same volume of 2X SDS loading buffer and boiled at 100 °C for 5 min, the supernatant was subjected to SDS-PAGE gel electrophoresis and blotting with anti-FLAG (Abcam, #ab49763) and anti-GFP (TransGen, #HT801) antibodies.

## 5. Conclusions

In this study, we found that constitutive expression of *EDS1* and *PAD4* simultaneously in *Arabidopsis* can enhance disease resistance at an elevated temperature, as well as at a normal temperature. To explore the potential application of this finding and evade the growth inhibition caused by constitutive overexpression of *EDS1* and *PAD4*, we generated transgenic *Arabidopsis* lines expressing both *EDS1* and *PAD4* under control of the *HSP70* promoter, whose expression is low at a normal temperature but could be highly induced at an elevated temperature. These transgenic lines exhibited enhanced resistance to *Pst* DC3000 at an elevated temperature without developmental defects. Thus, this study provided a new strategy for enhancing plant resistance to pathogens at an elevated temperature. The strategy could be potentially applied to breed disease-resistant crops.

## Figures and Tables

**Table 1:** Top 20 downregulated genes in *Arabidopsis* plants shifted from 28 to 19 °C identified from microarray dataset (GSE50019).

Gene ID	0 h-Average	24 h-Average	2 h-Average	8 h-Average	0 h vs. 24 h-FD	0 h vs. 2 h-FD	0 h vs. 8 h-FD
AT3G12580	3982.4	54.5	140.4	42.1	73.0	28.4	94.5
AT1G62510	3823.5	188.9	2727.7	52.1	20.2	1.4	73.4
AT5G12110	3711.1	186.1	551.7	87.9	19.9	6.7	42.2
AT3G28270	2741.2	130.0	595.2	57.4	21.1	4.6	47.8
AT5G48570	1333.9	17.8	69.0	15.2	74.9	19.3	87.8
AT5G12020	1093.1	14.9	128.0	12.5	73.4	8.5	87.6
AT5G52640	952.1	25.8	32.7	15.2	37.0	29.1	62.8
AT1G55260	833.1	114.6	619.2	404.2	7.3	1.3	2.1
AT1G60590	667.1	90.7	52.7	12.0	7.4	12.7	55.4
AT3G46230	512.1	17.1	52.4	12.5	29.9	9.8	40.8
AT1G17870	386.1	53.2	134.4	34.6	7.3	2.9	11.2
AT5G51440	372.0	10.3	14.1	9.7	36.1	26.3	38.2
AT4G26790	331.2	16.1	90.9	17.2	20.5	3.6	19.2
AT1G02205	304.0	15.4	478.5	39.6	19.7	0.6	7.7
AT2G29500	273.2	15.4	22.9	17.9	17.7	11.9	15.3
AT4G12400	270.0	11.4	15.6	10.4	23.8	17.3	26.0
AT1G72970	206.8	30.6	251.8	64.2	6.8	0.8	3.2
AT5G12030	200.2	9.7	9.8	9.2	20.6	20.3	21.7
AT1G07400	158.2	11.5	10.1	10.6	13.8	15.6	15.0
AT1G53540	120.0	13.5	22.2	14.1	8.9	5.4	8.5

**Table 2:** Top 10 downregulated genes in *Arabidopsis* plants shifted from 28 to 19 °C identified from RNA-seq dataset (GSE80448).

Gene ID	0 h-Average	4 h-Average	8 h-Average	0 h vs. 4 h_FD	0 h vs. 8 h_FD
AT5G48570	12139.3	1279.0	242.0	9.5	50.2
AT5G12110	11136.3	974.3	309.7	11.4	36.0
AT1G02205	3978.5	1111.8	608.7	3.6	6.5
AT3G12580	3647.3	368.7	14.0	9.9	260.5
AT5G52640	3336.3	218.3	22.7	15.3	147.2
AT4G12400	2415.5	114.3	22.7	21.1	106.6
AT1G60590	1182.2	260.0	27.5	4.5	43.0
AT1G17870	876.7	128.3	67.3	6.8	13.0
AT5G51440	620.3	82.7	6.0	7.5	103.4
AT4G26790	385.3	155.3	99.7	2.5	3.9

Average, average reads from 3 replicates. FD, foldchange.

## Data Availability

Not applicable.

## Supplementary Materials

The following are available online at https://www.mdpi.com/article/10.3390/plants10061258/s1, Figure S1: EDS1 and PAD4 protein accumulation in T1 transgenic lines. Table S1: Primers used in this study.
