# Supplementary material for: Temperature-Inducible Transgenic EDS1 and PAD4 in Arabidopsis Confer an Enhanced Disease Resistance at Elevated Temperature
Source: Plants (Basel). 2021 Jun 21;10(6):1258. doi: 10.3390/plants10061258 (PMC8234125; doi:10.3390/plants10061258)
Supplement: Supplementary file 1 [file plants-10-01258-s001.zip › plants-1252133-sup/Table S1.pdf]

Table S1. Primers used in this study.

Primer name	sequence 5'→3'	Purpose
AT4G26410- qF	GAGCGAAGAGGCACAATTC	qPCR
AT4G26410- qR	CTCTGCTAAAGCCGTCCT	qPCR
PR1-qF	TTCTTCCCTCGAAAGCTCAA	qPCR
PR1-qR	AAGGCCCACCAGAGTGTATG	qPCR
ICS1-qF	TACTAACCAGTCCGAAAGACG	qPCR
ICS1-qR	GAGGCTTGACAACAACTCTGT	qPCR
HSP70-qF	CACCGTCTTCGATGCTAAGC	qPCR
HSP70-qR	CTCAGCAGAGAACTGTTTCT	qPCR
AT5G12110-qF	TGGGGAGCTTCGAAATTGGT	qPCR
AT5G12110-qR	TCGACACTTTGGATGTACTC	qPCR
EDS1-qF	AGATTATTCAGGTGATCGAGCA	qPCR
EDS1-qR	TTTATGGGCTTGACACTTTGG	qPCR
PAD4-qF	TCAGTTAAAGATCAAGGAAGGA	qPCR
PAD4-qR	GGCGGAGAAGATTGAGATAGA	qPCR
proHSP70-L0-F	atgaagacaaggaggatcaggacttggggtttattg	Goldengate cloning
proHSP70-L0-R	gcgaagacaaatggtattagagatcagaattgttcgcc	Goldengate cloning
proAT5G12110-F	atgaagacaaggagagctcacagagagattatttgg	Goldengate cloning
proAT5G12110-R	gcgaagacaaatgggctcaagttaagtttctgtggaga	Goldengate cloning
