# Supplementary figures and images for: Temperature-Inducible Transgenic EDS1 and PAD4 in Arabidopsis Confer an Enhanced Disease Resistance at Elevated Temperature
Source: Plants (Basel). 2021 Jun 21;10(6):1258. doi: 10.3390/plants10061258 (PMC8234125; doi:10.3390/plants10061258)

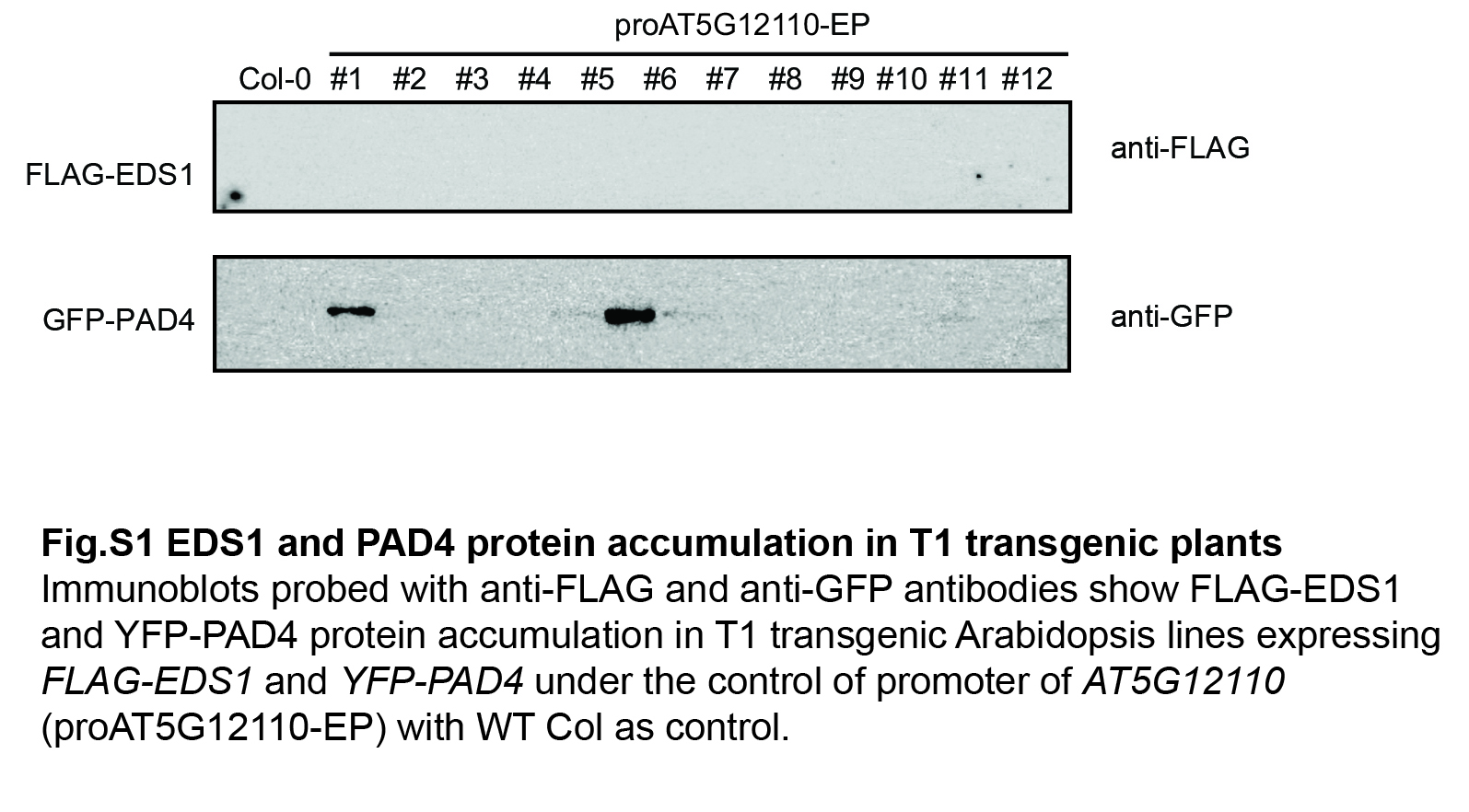

Supplement: Supplementary file 1 [file plants-10-01258-s001.zip › plants-1252133-sup/fig_S1.jpg]
